# Extra Copies of der(21)t(12;21) plus Deletion of *ETV6* Gene due to dic(12;18) in B-Cell Precursor ALL with Poor Outcome

**DOI:** 10.1155/2012/186532

**Published:** 2012-03-25

**Authors:** Marina Araújo Fonzar Hernandes, Terezinha de Jesus Marques-Salles, Hasmik Mkrtchyan, Eliane Maria Soares-Ventura, Edinalva Pereira Leite, Maria Tereza Cartaxo Muniz, Maria Teresa Marquim Nogueira Cornélio, Thomas Liehr, Neide Santos, Maria Luiza Macedo Silva

**Affiliations:** ^1^Pediatric Oncohematology Center, Hospital Oswaldo Cruz, University of Pernambuco (CEONHPE/HUOC/UPE), 50050-210 Recife, PE, Brazil; ^2^Institute of Biological Science, University of Pernambuco, 50050-210 Recife, PE, Brazil; ^3^Departament of Genetics, Federal University of Pernambuco, 50670-901 Recife, PE, Brazil; ^4^Institute of Human Genetics and Anthropology, 07740 Jena, Germany; ^5^Department of Cytogenetics of the National Center for Bone Marrow Transplant (CEMO-INCA), National Cancer Institute, 20230-130 Rio de Janeiro, RJ, Brazil

## Abstract

Acute lymphoblastic leukemia (ALL), CD10+ B-cell precursor, represents the most frequent type of childhood ALL from 3 to 6 years of age. The t(12;21)(p13;q22) occurs in 25% of cases of B-cell precursor ALL, it is rare in children less than 24 months and have been related to good prognosis. Some relapse cases and unfavorable prognosis in ALL CD10+ are associated with t(12;21) bearing additional aberrations as extra copies of chromosome 21 and *ETV6* gene loss. This report describes the case of a 15 month-year old girl, who displayed a karyotype with addition on chromosome 12p plus trisomy 10 and tetrasomy of chromosome 21. Molecular cytogenetic studies revealed two extra copies of the der(21) t(12;21), trisomy 10 and deletion of the second *ETV6* gene due to the dic(12;18). These findings show the great importance of molecular cytogenetic studies to clarify complex karyotypes, to define prognostic, to carry out risk group stratification and to support correctly disease treatment in childhood acute lymphoblastic leukemia.

## 1. Introduction

While infant acute lymphoblastic leukaemia (ALL) accounts for about 3% of leukemias in children below 12 months, it displays specific clinical characteristics with most cases having phenotype pro-B, 80% of cases presenting *MLL *gene rearrangement, and represents a group of poor prognosis [1]. The ALL cases, CD10+ B-cell precursor (BCP), have the incidence peak among 3–6-year olds and are considered the most frequent (85–90%) type of childhood leukemia with good prognosis [2]. The characteristic cytogenetic abnormality is the classic “cryptic” translocation t(12;21)(p13;q22), defined also as *ETV6/RUNX1 *(*TEL-AML1*) gene fusion, detected by fluorescent *in situ* hybridization (FISH) in 20–25% of cases [3, 4].

In CD10+ B-cell precursor (BCP) ALL, translocations or dicentrics chromosomes involving 12p are mostly associated with loss of 12p material and a lot of partners chromosomes are described. Cases with these abnormalities are rarely described in children younger than 24 months [5]. Chromosome 12 breakpoint is most often localized in 12p13, involving *ETV6* gene, with fusion of 5′ end of *ETV6* with 3′ end of the partner and sometimes accompanied with a concomitant deletion of other *ETV6* allele gene [5]. In B-cell precursor (BCP) ALL some reports have shown that patients with t(12;21) and secondary abnormalities such as complex karyotypes, extra copy of der(21)t(12;21), gain of one or two *RUNX1 *allele, or deletion of second *ETV6* allele gene have a poor prognosis [5, 6].

 Here a case of a 15-month-year old girl is reported in which conventional cytogenetic analysis revealed addition on chromosome 12p plus trisomy 10 and tetrasomy of chromosome 21. Molecular cytogenetic studies have revealed trisomy 10 plus two extra copies of the der(21)t(12;21) and deletion of the second *ETV6* gene due to the dic(12;18). The infant did not respond to chemotherapy and consequently died due to disease progression.

## 2. Materials and Methods

### 2.1. Case Report

The 15-month old girl admitted at the Pediatric Oncohematology Center, University Hospital Oswaldo Cruz (Recife, Pernambuco) with history of anaemia and bone pain. The child showed pallor, petechiae, microadenopathy, and hepatomegaly. The blood peripheral examination revealed the following: Hg = 9,8 g/dL; WCB = 22.629/mm³ (91% lymphoblast), platelets count = 21.000/mm³. The bone marrow was hypercellular with 90% infiltration by blast ALL-L1. Immunophenotyping studies showed the following: CD45+; TdT+; HLA-Dr+; CD22+, CD19+; CD10+. RT-PCR analysis demonstrated the presence of *ETV6/RUNX1* transcripts. The child was treated with high-risk GBTLI-99 ALL protocol and relapsed after 17 months from diagnosis. At relapse she was treated with BFM-90 ALL protocol, but died due to progressive disease.

### 2.2. Conventional Cytogenetic and Molecular Analysis

Conventional cytogenetic analysis was performed on unstimulated bone marrow cells cultivated for 24 hours. G-banding was done according to standard protocol. Karyotype was described according to ISCN (2009) [7]. FISH analysis with LSI MLL dual colour, break-apart rearrangement probe (Vysis UK), and LSI TEL/AML1 ES dual colour, extra signal translocation probe (Vysis UK) were used according to the manufacturer's instructions. At least 100 interphase nuclei were analyzed in each study. Multicolor FISH (M-FISH) using 24 whole chromosome painting probes, FISH with CEP12 and CEP18 were applied and multicolor banding (MCB) was performed as previously described [8, 9].

## 3. Results

**Figure 1:**
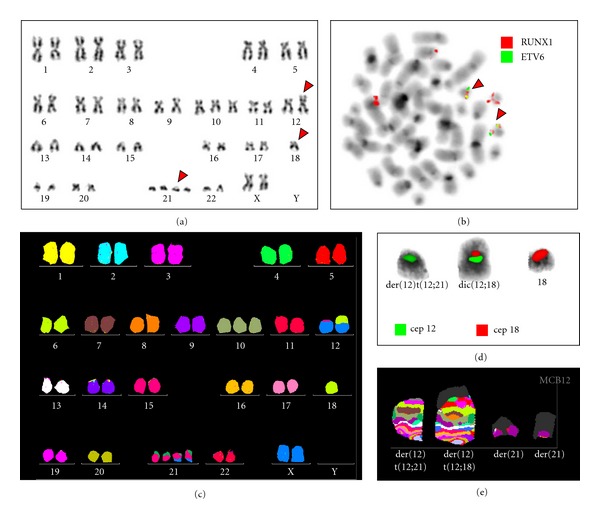
Conventional and molecular cytogenetics dates. (a) G-banding. The arrows show add (12p), −18, and +21, +21; (b) FISH shows two copies of der(21)t(12;21) (arrows), two 21 chromosomes, and *ETV6* deletion; (c) M-FISH showing the 10 trisomy and der(12)t(12;21); dic(12;18)(p12;q11.21), der(21)t(12;21), +21; (d) CEP probe 12 and 18 shows dic(12;18); (e) MCB: showing the breakpoints of the der(12)t(12;21) and der(12)t(12;18).

Cytogenetic study by G-banding revealed a complex karyotype 48, XX, +10, add(12p), −18, +21, +21[7]/46, XX[13] (Figure  1(a)). FISH showed no rearrangements to* MLL* gene, and with dual colour probe LSI TEL/AML1 ES identified the *ETV6/RUNX1* fusion with extra copy of der(21)t(12;21), extra chromosome 21, and deletion of second *ETV6* gene in 50% of interphase nuclei (Figure  1(b)). To better characterize the karyotype M-FISH (Figure  1(c)) and MCB for chromosomes 12 and 18 were performed. MCB showed a translocation involving the regions 12p12 and 18p11.21 and FISH with CEP probes confirmed unmasked dic(12;18) (Figures 1(d)–1(e)). The final karyotype was characterized as 48, XX, +10, t(12;21)(p13,q22), dic(12;18)(p12;q11.21), −18, +der(21)t(12;21), +21[10]/46, XX[10].

## 4. Discussion

Several studies have investigated the prognosis value of t(12;21) positive ALL, but in general it is associated with a good prognosis. However, a few cases present relapsed (10–20%) [10]. The heterogeneity of clinical response seems to depend on the intensity of treatment and additional genetic changes [11].

The case related here, a BCP-ALL with t(12;21) that presented extra copy of der(21)t(12;21) and a deletion of the second *ETV6* gene due to the dic(12;18) plus extra copy, had a progressive disease despite the aggressive treatment. All those aberrations when found are rare and described in only 1% of t(12;21) positive ALL [11]. The favourable prognosis of translocation t(12;21) seems to be impaired by the presence of extra aberrations [12–14]. The extra copy der(21) has been reported to be more frequently in relapsed case and the presence at diagnosis is linked to an unfavourable prognosis [15]. Whether other abnormalities, such as deletion of *ETV6* and extra copy of *RUNX1* also, contribute to unfavourable prognosis is still controversial [16, 17]. However, the *ETV6* gene deletion in childhood ALL is discussed as a hint on a tumour suppressor gene function [18].

 The case described here showed the great importance of molecular cytogenetic studies to clarify the cryptic translocations and marker chromosomes, mainly to define prognostic, risk group stratification and to provide appropriate support in treating the acute lymphoblastic leukemia. Other cases are needed in order to define whether those aberrations should be treated only with chemotherapy or, as in some MLL rearrangements cases, the bone marrow transplantation should be part of the first remission treatment.
